# calf – Software for CEST Analysis with Lorentzian Fitting

**DOI:** 10.1007/s10916-023-01931-6

**Published:** 2023-03-24

**Authors:** Hans-Jörg Wittsack, Karl Ludger Radke, Julia Stabinska, Alexandra Ljimani, Anja Müller-Lutz

**Affiliations:** 1 Medical Faculty, Department of Diagnostic and Interventional Radiology, University Dusseldorf, D-40225 Dusseldorf, Germany; 2 F.M. Kirby Research Center for Functional Brain Imaging, Kennedy Krieger Institute, Baltimore, MD 21205 USA; 3 Division of MR Research, The Russell H. Morgan Department of Radiology and Radiological Science, The Johns Hopkins University School of Medicine, Baltimore, MD 21205 USA

**Keywords:** MRI, CEST analysis, z-spectra, Lorentzian fitting, Software

## Abstract

Analysis of chemical exchange saturation transfer (CEST) MRI data requires sophisticated methods to obtain reliable results about metabolites in the tissue under study. CEST generates z-spectra with multiple components, each originating from individual molecular groups. The individual lines with Lorentzian line shape are mostly overlapping and disturbed by various effects. We present an elaborate method based on an adaptive nonlinear least squares algorithm that provides robust quantification of z-spectra and incorporates prior knowledge in the fitting process. To disseminate CEST to the research community, we developed software as part of this study that runs on the Microsoft Windows operating system and will be made freely available to the community. Special attention has been paid to establish a low entrance threshold and high usability, so that even less experienced users can successfully analyze CEST data.

## Introduction

Chemical exchange saturation transfer (CEST) MRI is an evolving metabolic imaging method, which has proven as valuable in many imaging studies in the last two decades [1–3]. In particular, CEST has been shown to add diagnostic and prognostic value to the conventional MRI in patients with cerebrovascular stroke, neurological diseases, tumor imaging, and musculoskeletal disorders [4]. Despite its potential to provide unique metabolic information [5–10], CEST is not yet widely used in the clinical settings. Two major reasons for this are: firstly, the limited availability of CEST acquisition sequences on clinical MRI systems and, secondly, the relatively high complexity of CEST data analysis. To facilitate the clinical translation of CEST MRI, we present new methods for CEST data processing and analysis using the Lorentzian fitting approach. The methods are implemented in a user-friendly software that runs on the Microsoft Windows operating system, and are made available to the CEST imaging community.

In CEST MRI multiple images are acquired after applying radio-frequency (RF) saturation pulses at several frequency offsets upfield and downfield from the bulk water resonance [2]. By point plotting of the image intensity as a function of the offset frequency of the pre-saturation, the so-called z-spectra are obtained pixel by pixel. The frequency selective irradiation ahead of the image acquisition causes a pre-saturation of the labile protons on functional groups of metabolites. Exchanging protons from these individual molecular groups transfer their magnetization to water protons, leading indirectly to a decrease of the measured water signal intensity. This saturation transfer process creates signal peaks that exhibit a Lorentzian line shape [11, 12] with a center frequency offset specific for each functional group.

The acquisition process based on an alternating repetition of the pre-saturation and image recording is time-consuming, resulting in z-spectra with a limited number of data points in frequency direction. Besides, the necessary fast image acquisition usually yields a relatively low signal-to-noise ratio (SNR), leading to noisy z-spectra [13]. This is especially problematic as the CEST effect usually results in waters signal intensity changes that are only a few percent. Finally yet importantly, the resonance frequency in MRI is proportional to the external magnetic field, leading to a substantial shift of the z-spectra in frequency direction even at low levels of static magnetic field inhomogeneities [14, 15].

To cope with these problems in the analysis of the z-spectra, different approaches and corrections were proposed. The most common and rather qualitative metric is the magnetization transfer ratio asymmetry (MTR_asym_) [16, 17], which is defined as the difference between the z-spectrum value at the negative offsets and corresponding positive offsets with respect to water. In asymmetric spectra due to the nuclear Overhauser effect (NOE) or other confounding effects, MTR_asym_ has a restricted explanatory power, especially in cases where the measured CEST effect is low or in molecules resonating close to the water resonance frequency. To improve the analysis of z-spectra, mathematical fitting using Lorentzian model functions was established [18–22].

In this method, each component of the z-spectrum – the water component and all other resonances from exchangeable protons in different functional groups of e.g. amide, amine, creatine, and NOE – is represented by a Lorentzian line, added up to the complete z-spectrum. Each Lorentzian line exhibits three free parameters: amplitude, frequency, and line width. Consequently, modelling the measured z-spectra with multiple Lorentzian’s leads to a relatively high number of fitting parameters compared to the low number of data points acquired in frequency direction. The simplest method of fitting complex model functions to measured data is the nonlinear least squared (NLLS) technique, such as the Levenberg-Marquard algorithm. However, more adapted least squared methods were developed and established to analyze sparse data with low SNR. For example, methods such as VARPRO or AMARES have been proposed for the in-vivo MR-spectroscopy (MRS), where -similar to CEST- low SNR signals with strongly overlapping resonances occur [23, 24]. However, while the MRS dataset typically consists of 1000 or more data points, the CEST z-spectrum contains relatively few data points in the frequency direction (e.g., about 40 points). Further, due to the indirect nature of the method, the individual resonances of the molecules or functional groups often strongly overlap. Therefore, high-quality technology is required to analyze such complicated signals.

Here we present a sophisticated method based on an adaptive nonlinear least squares algorithm, which allows a robust quantification of the z-spectra and includes prior knowledge in the fitting process. The software developed as a part of this study runs on the Windows 10 operating system and is made freely available to the community. Special attention has been paid to establish a low entrance threshold and high usability, so that even less experienced users can successfully analyze CEST data.

In our study, we first show the theoretical background of the developed software. Simulated data were then created and analyzed to validate the algorithms, taking advantage of the great benefit of known ground truth. Finally, to demonstrate the suitability of the new software, CEST data from a real phantom and the lumbar spine of a healthy subject were analyzed.

## Methods

### Fitting of Lorentzians

Any z-spectrum can be modeled by the sum of several Lorentzian functions described by the following function:

1 $$L\left({a}_{k},{\omega }_{k}^{c},{\sigma }_{k}\right)= 1-\frac{I}{{I}_{0}}=\sum_{k=1}^{K}\frac{{a}_{k}}{1+4{\left(\frac{\omega -{\omega }_{k}^{c}}{{\sigma }_{k}}\right)}^{2}}$$

where $I$ is the image intensity, ${I}_{0}$ is the image intensity without pre-saturation, $K$ is the number of Lorentzian components, $\omega$ is the frequency and ${a}_{k}$, ${\omega }_{k}^{c}$ and ${\sigma }_{k}$ are the amplitude, frequency offset and width of the $k$ ^th^ CEST proton pool.

According to probability theory, to obtain maximum likelihood estimates, the following functional $\Phi$ must be minimized:

2 $$\Phi \left({\varvec{a}},{{\boldsymbol{\omega}}}^{{\varvec{c}}},{\boldsymbol{\sigma}}\right)=\sum_{i=0}^{N-1}{\left|{y}_{i}- \sum_{k=1}^{K}\frac{{a}_{k}}{1+4{\left(\frac{{\omega }_{i}-{\omega }_{k}^{c}}{{\sigma }_{k}}\right)}^{2}}\right|}^{2}={\Vert {\varvec{y}}- \Psi {\varvec{a}}\Vert }^{2}$$

where ${\varvec{y}}={\left[{y}_{0},{y}_{1},{y}_{2},\dots {y}_{N-1}\right]}^{{\varvec{T}}}$ is the signal vector, $N$ is the number of frequency offsets measured, ${\varvec{a}}={\left[{a}_{0},{a}_{1},{a}_{2},\dots {a}_{K}\right]}^{{\varvec{T}}}$ is the amplitude vector, ${{\boldsymbol{\omega}}}^{{\varvec{c}}}={\left[{\omega }_{1}^{c},{\omega }_{2}^{c},{\omega }_{2}^{c},\dots {\omega }_{K}^{c}\right]}^{{\varvec{T}}}$ is the frequency vector and ${\boldsymbol{\sigma}}={\left[{\sigma }_{1},{\sigma }_{2},{\sigma }_{3},\dots {\sigma }_{K}\right]}^{{\varvec{T}}}$ is the width vector of the Lorentzian components. $\Psi$ is the following $N x K$ matrix of full rank:

3 $$\Psi =\left[\begin{array}{ccc}\frac{1}{1+4{\left(\frac{{\omega }_{0}-{\omega }_{1}^{c}}{{\sigma }_{1}}\right)}^{2}}& \dots & \frac{1}{1+4{\left(\frac{{\omega }_{0}-{\omega }_{K}^{c}}{{\sigma }_{K}}\right)}^{2}}\\ \vdots & \ddots & \vdots \\ \frac{1}{1+4{\left(\frac{{\omega }_{N-1}-{\omega }_{1}^{c}}{{\sigma }_{1}}\right)}^{2}}& \dots & \frac{1}{1+4{\left(\frac{{\omega }_{N-1}-{\omega }_{K}^{c}}{{\sigma }_{K}}\right)}^{2}}\end{array}\right]$$

The superscript T denotes the transpose and $\Vert \dots \Vert$ the Euclidian norm.

The functional $\Phi$ consists of the sum of squared residuals and leads to a typical nonlinear least squared problem. To solve such problems, Dennis et al. developed an adaptive NLLS algorithm that can handle large residuals or very nonlinear problems better than a Levenberg–Marquardt algorithm [25–27]. In our implementation, we used the subroutine *dn2gb* from the *port* library of *netlib* [28], an established collection of mathematical software commonly used in science and engineering. By applying upper and lower bounds in *dn2gb*, prior knowledge such as large linewidths for magnetization transfer components or known frequencies of CEST metabolites can be included in the fitting process. Adding such physical requirements as additional bounds leads to maximum accuracy and robustness [29]. As a secant method, the NLLS algorithm applied here uses the evaluation of the residuals and the Jacobi matrix $J$, which consists of the first derivatives of the residuals according to the unknown parameters:

4 $$\begin{array}{l}\frac{{\partial J}_{i,k}}{\partial {a}_{k}}= \frac{1}{1+4{\left(\frac{{\omega }_{i}-{\omega }_{k}^{c}}{{\sigma }_{k}}\right)}^{2}}, \\ \frac{{\partial J}_{i,k}}{\partial {\omega }_{k}}= \frac{8{a}_{k}({\omega }_{i}-{\omega }_{k}^{c})}{{{\sigma }_{k}^{2}\left[1+4{\left(\frac{{\omega }_{i}-{\omega }_{k}^{c}}{{\sigma }_{k}}\right)}^{2}\right]}^{2}}, \\ \frac{{\partial J}_{i,k}}{\partial {\sigma }_{k}}=\frac{8{a}_{k}{({\omega }_{i}-{\omega }_{k}^{c})}^{2}}{{{\sigma }_{k}^{3}\left[1+4{\left(\frac{{\omega }_{i}-{\omega }_{k}^{c}}{{\sigma }_{k}}\right)}^{2}\right]}^{2}}\end{array}$$

### Software development

To create an easy to use tool for CEST analysis, the proposed software was developed under Windows 10 operating system in Microsoft Visual C++ 2022 using the MFC library as graphical user interface. The usual design elements of a typical Windows software have been used so that users can easily and quickly find their way around. Since the *netlib* library was developed in the Fortran programming language, *dn2gb* was compiled with the Intel® Fortran compiler *ifort v.2021.6.0*. Then the corresponding lib-file was created using Microsoft Library Manager, which is part of Visual Studio. Fortran passes all arguments of subroutines by reference. Therefore, pointers must be used when calling Fortran subroutines from C++. Data structures like vectors, matrices and arrays are represented in different ways in Fortran and C++. Consequently, when calling the external Fortran subroutines from C++, special care must be taken with the construction and order of the variables.

### Filtering and pyramidal approach

The SNR of CEST data is a relevant aspect to be considered. For this reason, two approaches were proposed to improve the stability of the results. The first one uses image filtering prior to z-spectra generation and analysis, whereas the second one is based on a pyramidal approach similar to the Image Dowsampling Expedited Adaptive Least-squares (IDEAL) method presented by Zhou et al. [12]. For noise filtering in image space, a non local means (NLM) filter [30] was implemented using the OpenCV 4.5.1 library [31]. Compared to simple Gaussian filters, which cause significant image smoothing, NLM filters preserve image details. In the pyramidal method, image downsampling is used to increase SNR. First, the image size of the original image data is strongly reduced by a factor of 2^N^, where N is a small integer number. Next, the averaged signal of the down-sampled images is analyzed by Lorentzian fitting and the obtained results are then extrapolated by a factor of two and serve as initial values for analyzing the up-sampled images. The up-sampling steps are repeated until the original image resolution is reached. Zhou et al. demonstrated that this approach leads to more robust results both in phantom and rat brain data [12].

### CEST analysis

Due to the inhomogeneity of the static magnetic field B_0_, the center frequency of the water peak, which is normally at 0 ppm, is often shifted. Depending on the inhomogeneity of B_0_, the shift can exceed 1 ppm [32]. When analyzing the z-spectra by Lorentzian fitting, B_0_ shift is less relevant as long as the fitted Lorentzian components can be assigned to the individual proton pools. This means that corrections of the B_0_ inhomogeneity by pixel-wise shifting of the z-spectrum, e.g. with the water-shift referencing (WASSR) method [14], are not necessary for the Lorentzian fitting because no asymmetry analysis is performed. Nevertheless, our CEST analysis performed frequency correction by pre-fitting to obtain reliable frequency offsets for the individual components. It should be noted that this water shift step only serves to facilitate the localization of the various CEST components, but does not affect the results of the fitted lines as long as the fitting is successful. In this initial step, a sum of six Lorentzian’s is fitted to each z-spectrum to determine the center of the water resonance. The fitting is loosely constrained and starts with a large peak for water at 0 ppm, two peaks above and below the water peak at ± 1.0 ppm and ± 2.0 ppm and a broad peak at -2.0 ppm representing possible semisolid magnetization transfer (MT) contrast in the spectrum. The resulting frequency of the fitted water peak is then used to shift the original z-spectra, and the actual fitting analysis starts. This allows easy assignment of the individual components to the known frequencies.

In the actual analysis of the z-spectra, a set of starting values is introduced to the fitting process. This set contains the number of Lorentzian components, the frequency range swept by RF saturation pulses, and three parameters for each component, namely the amplitude, frequency offset, and line width. For the latter three parameters, constraints are given by lower and upper bounds, restricting the possible solutions for each individual Lorentzian line. In this way, prior physical knowledge is incorporated into the fitting process. When analyzing CEST images with the pyramid method, it is possible to select the number of pyramid levels and a tolerance value in percent that specifies a range within which the parameters are allowed to change from one pyramid level to the next.

The fitting process of the z-spectra yields the optimal amplitude, frequency and width for each individual Lorentzian line, the integral of each component, and the sum of squares between the fitting result and the original z-spectra as a quality measure.

### Creation of digital phantoms

To evaluate the newly developed algorithms and software, two digital phantoms with different compositions of proton pools were created. The advantage of a digital phantom is the known Lorentzian composition as a ground truth. The phantoms were overlaid pixel by pixel with Rice-distributed noise of varying amplitude. The noise amplitude ranged from 0–20% in the image without pre-saturation, resulting in ideal z-spectra without noise and “more realistic” z-spectra of different quality.

**Fig. 1:**
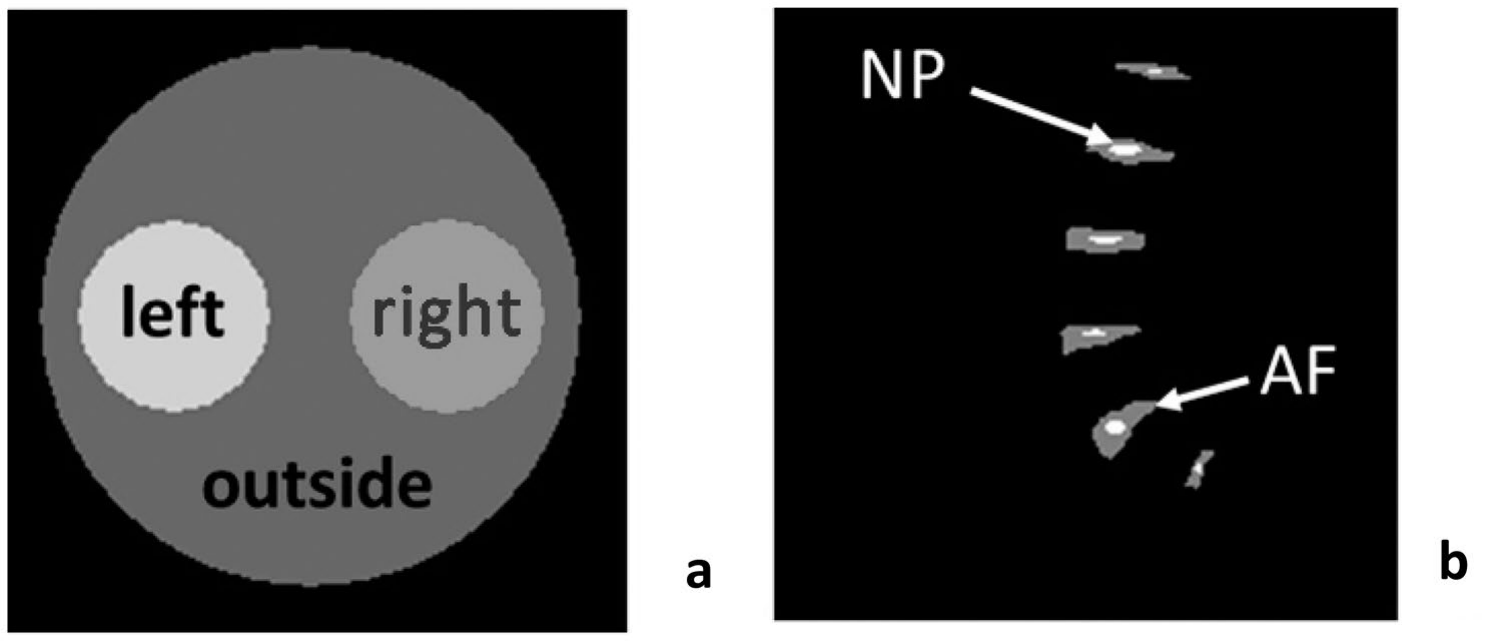
Composition of digital phantoms. **a** Phantom #1 consisted of three regions: left and right circle and outer region. Various amounts of metabolites were added within the small circles, while only water was present in the outer area. **b** Phantom 2# consists of a structure of lumbar intervertebral discs with two different regions: Nucleus pulposus and annulus fibrosus

A cylindrical structure with two inner circles was chosen as the first phantom, as shown in Fig. 1a. The outer region of the phantom consisted entirely of a water pool, and the left and right inner regions contained a composition of water (0 ppm), creatine (1.9 ppm), amide (3.5 ppm), OH (1.0 ppm) and MT (–2.0 ppm) pools. The amide and creatine amplitudes varied between the left and right inner regions, while all other components remained constant. The complete composition is shown in Table 1.

**Table 1:** Composition of digital phantom #1. Amide and creatine amplitude varied between the left and right regions, while all other pools remained constant. The outer region consisted of water only

Proton pool	Amplitude (I/I_0_) left	Amplitude (I/I_0_) right	Amplitude (I/I_0_) outside	Frequency [ppm]	Width [ppm]
H_2_O	0.95	0.95	0.95	0.0	0.5
Amide	0.30	0.10	0.00	3.5	0.5
Creatine	0.10	0.30	0.00	1.9	0.5
OH	0.03	0.03	0.00	1.0	0.5
MT	0.03	0.03	0.00	–2.0	15.0

In addition, a second digital phantom was created with a structure similar to that of a sagittal spine image in which only the intervertebral discs (IVDs) were preserved (see Fig. 1b). Since this model is typical for CEST studies of glycosaminoglycans (GAG), two regions were defined in each disc, representing the inner nucleus pulposus (NP) and the outer annulus fibrosus (AF). The composition of the two regions was water (0 ppm), GAG (OH) (1.0 ppm), NH (3.2 ppm), MT (–2.43 ppm), NOE (–2.6 ppm) and NOE (–1.0 ppm). The amplitude of the GAG component varied between NP and AF and was greater in NP. The complete composition is shown in Table 2.

**Table 2:** Composition of digital phantom #2. The GAG (OH) amplitude varied between AF and NP, while all other pools remained constant

Proton pool	Amplitude (I/I_0_) AF	Amplitude (I/I_0_) NP	Frequency [ppm]	Width [ppm]
H_2_O	0.75	0.75	0.0	2.0
GAG (OH)	0.03	0.06	1.0	1.0
NOE-2.6	0.05	0.05	–2.6	1.0
NOE-1.0	0.001	0.001	–1.0	2.0
NH	0.03	0.03	3.2	0.5
MT	0.15	0.15	–2.4	10.0

### Validation of the software

To validate our software, the digital phantoms were overlaid with simulated noise and systematically analyzed. Noise with amplitudes from 0 to 20% for Phantom 1 with large geometric structures and from 0 to 10% for Phantom 2 with small geometric structures was superimposed in steps of 1%. Since the image noise severely reduces the visibility of small structures, a smaller amount of noise was added in Phantom 2. The resulting CEST images were analyzed in three ways: without any pre-processing (normal), after applying the NLM filter, and using the pyramidal approach (pyramid). In Phantom 2, a combined method of NLM and pyramidal approach was also used to further improve the CEST analysis. The pixel-by-pixel Lorentzian fitting resulted in parametric images of amplitudes, frequency offsets and widths. Further, integral images for each individual proton pool as well as the sum of squares maps were created. All the calculated parameter maps were analyzed within the predefined regions with different proton pool compositions. The mean and standard deviation of the regions were analyzed as a function of the added image noise. In addition, the standard deviations of the predefined regions were compared between the three approaches: normal, NLM and pyramid. Besides, the regional sum of squares for the three different methods were compared as a quality measure for the fit analysis.

#### Phantom

To validate the algorithms and software for analyzing measured MRI data, a phantom containing different amounts of creatine (Cr) and nicotinamide (NAD) was created. This water-based phantom consisted of four inner tubes filled with NAD and Cr dissolved in phosphate-buffered saline (ROTI®Cell PBS, Carl ROTH) at concentrations ranging from 50 to 100 mM (Table 3), and physiological pH of 7.3.

**Table 3:** Composition of the phantom for real MRI measurements. The phantom consisted of 4 inner tubes with different concentrations of amide (NAD) and creatine (Cr)

Tube	#1	#2	#3	#4
NAD	100 mM	100 mM	50 mM	50 mM
Cr	50 mM	100 mM	100 mM	50 mM

The acquired CEST data from the phantom were preprocessed by NLM filter to increase the SNR before analysis by Lorentzian fitting.

#### In-vivo study

To test the utility of the developed software, an in-vivo study was performed on a healthy volunteer. CEST data were acquired from the lumbar spine. Based on the results of the digital phantom #2, the CEST data of the spine were processed using the NLM and pyramidal approach. The study was approved by the local ethics committee (Ethics Committee of the Medical Faculty of Heinrich Heine University, Düsseldorf, Germany). Written informed consent was obtained from healthy volunteers who participated this study.

### MRI measurements

All acquisitions were performed on a Siemens Magnetom Prisma MRI (Siemens Healthineers, Erlangen, Germany) at a magnetic field strength of 3 T. The phantom experiment was performed with an eight channel knee coil. A 24-channel spine coil was used for the lumbar spine acquisition. CEST was acquired with an in-house developed spoiled gradient echo sequence using the following parameters: repetition time TR = 7.2 ms, echo time TE = 3.5 ms, excitation flip angle = 15°, image matrix = 128 × 128, slice thickness = 10 mm (phantom), 6 mm (in-vivo), field of view = 80 × 80 mm^2^ (phantom), 200 × 200 mm^2^ (in-vivo), 1 pre-saturation pulse (phantom), 40 pre-saturation pulses (in-vivo), B_1_ = 1.5 μT (phantom), 0.9 μT (in-vivo), pulse duration of PD = 900 ms (phantom), 100 ms (in-vivo), frequency offsets = 63, frequency range = –7.0– 7.0 ppm (phantom), –5.0–5.0 ppm (spine).

## Results

### Software development

**Fig. 2:**
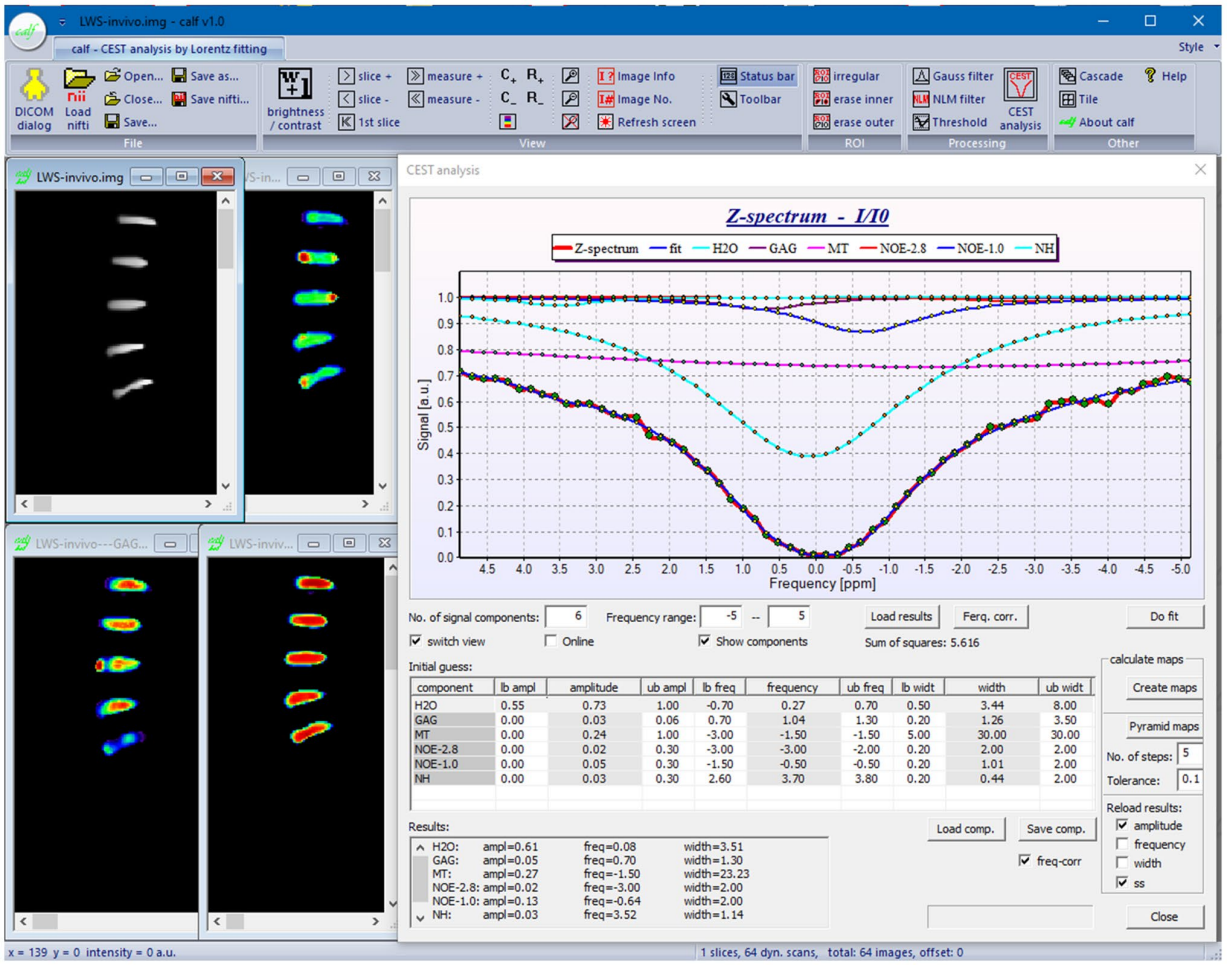
Screenshot of the “calf” software, showing the graphical user interface with the standard Windows ribbon toolbar. On the left, CEST data of an in-vivo examination of the lumbar spine are shown. On the right, the z-spectrum of an intervertebral disk with the fitted components are shown as an example

With “calf”, a user friendly software has been developed that enables the analysis of CEST data with an arbitrary number of proton pools, each represented by a Lorentzian line. The software runs on Microsoft Windows operating system, has a graphical user interface, and is easy to use (see Fig. 2). The software reads DICOM and Nifti as input image formats. “calf” allows navigation through 4D image data and display of z-spectra of manually selected regions of interest. Users can define arbitrary CEST proton pools at defined offset frequencies as prior knowledge for the analysis process. Constraints can be inserted for each component to stabilize the mathematical fitting process. As shown in Fig. 2, a table of initial values and constraints for each proton pool can be passed to the software. This gives the user the freedom to adapt the fitting to his needs. In particular, different numbers of proton pools and different compositions of metabolites or molecular groups can be considered. Tables created for specific applications, such as APT-CEST or gagCEST, can be saved and shared between users. The software analyzes single z-spectra or whole CEST image data and provides parameter images of amplitude, width and frequency of each predefined proton pool. To reduce image noise within the CEST data, a simple Gaussian filter and NLM filter have been implemented for the calculation of parameter images. Furthermore, a pyramidal approach is available to improve results for CEST parameter images. "calf" is available to the CEST community and can be downloaded from https://github.com/MPR-UKD/calf.

### Digital phantoms

To illustrate the software evaluation process the results of phantom 1 and 2 are presented below. After presenting z-spectra and CEST parameter maps of phantom 1 at single noise values as an example, the effects of NLM noise filter and pyramidal approach are shown. Finally, the quantitative fitting results of phantom 1 as a function of image noise are presented and compared to the ground truth of the simulated CEST data. Since the digital phantom 2 has a different CEST composition and geometric shape, the CEST results are then presented again as a function of image noise. Finally, the quantitative results of the GAG component of phantom 2 are presented as a function of image noise.

**Fig. 3:**
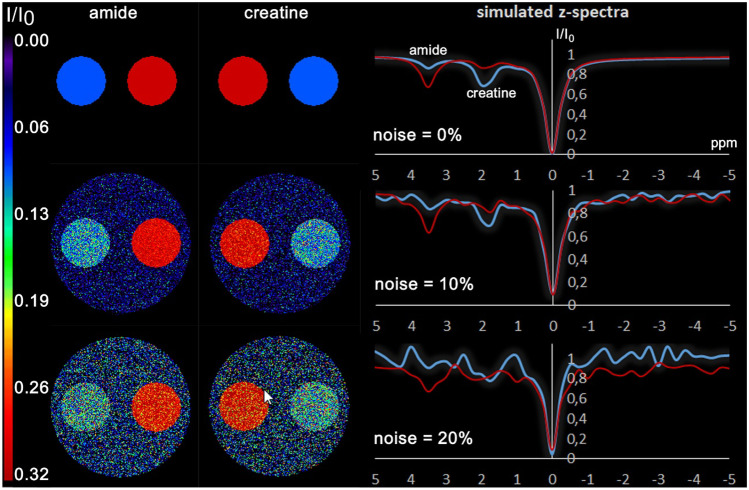
Digital phantom #1. On the right exemplary z-spectra of the right (red) and left (blue) inner circle are shown at noise levels 0, 10% and 20%. On the left the corresponding Lorentzian-based amplitude maps of the amide and creatine peaks are displayed

Figure 3 shows the digital phantom 1 with corresponding exemplary z-spectra of the right and left inner circles at three noise levels. In the spectra without noise, the resonances of amide and creatine are clearly visible, while at low SNR the peaks at 3.5 ppm and 2.0 ppm are difficult to distinguish from the signal noise. The parametric maps of amide and creatine show exactly the amplitude of the ground truth shown in Table 1 for the case without noise. The more noise is added to the phantom, i.e. the z-spectra, the noisier are the resulting parametric images.

**Fig. 4:**
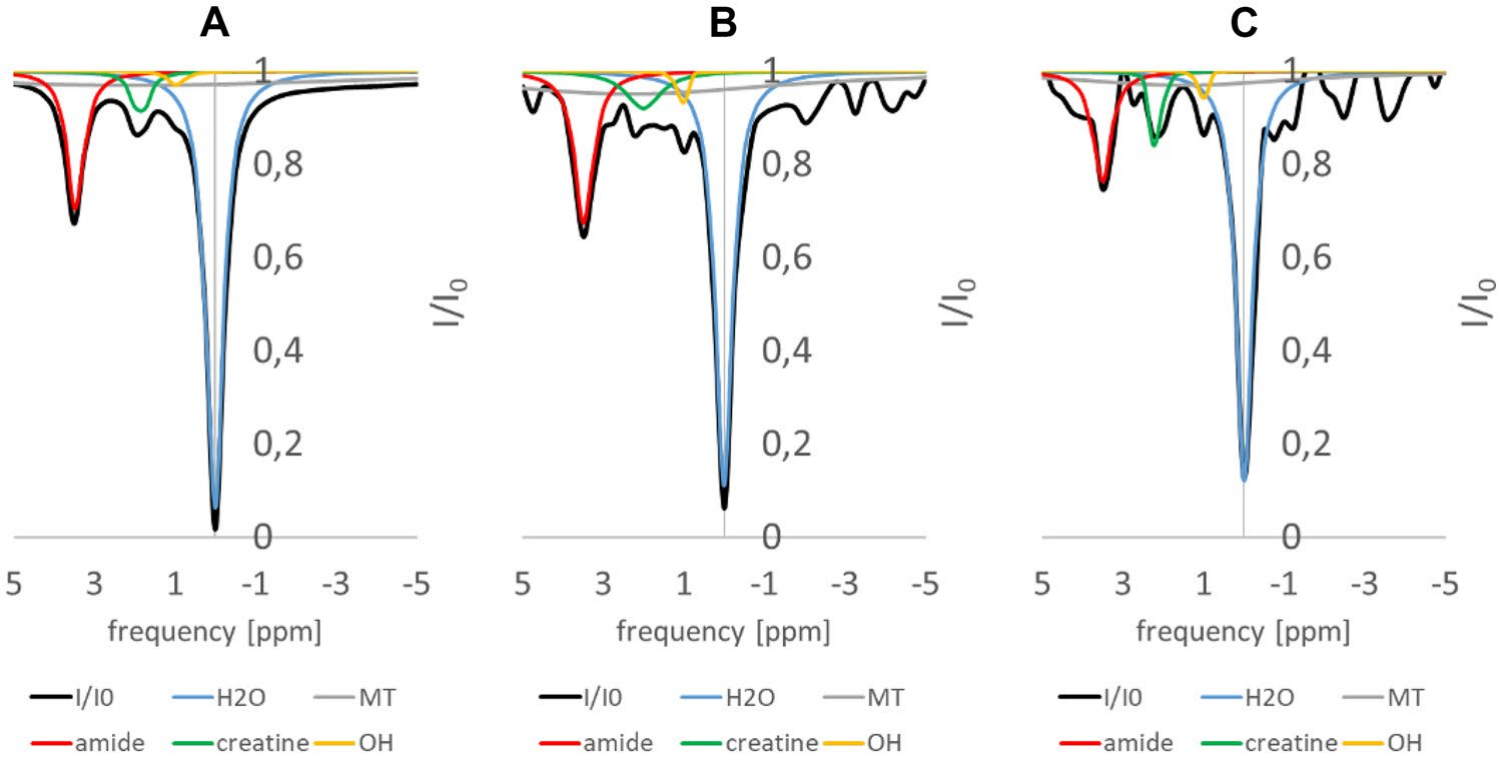
Exemplary z-spectra of phantom #1 at three different noise levels (**A** = 0%, **B** = 10%, **C** = 20% noise) including the fitted CEST components

Figure 4 shows three exemplary z-spectra of phantom 1 at different noise levels including the fitted CEST components (see Table 1). Notably, even at high noise levels, the spectrum is accurately modeled.

**Fig. 5:**
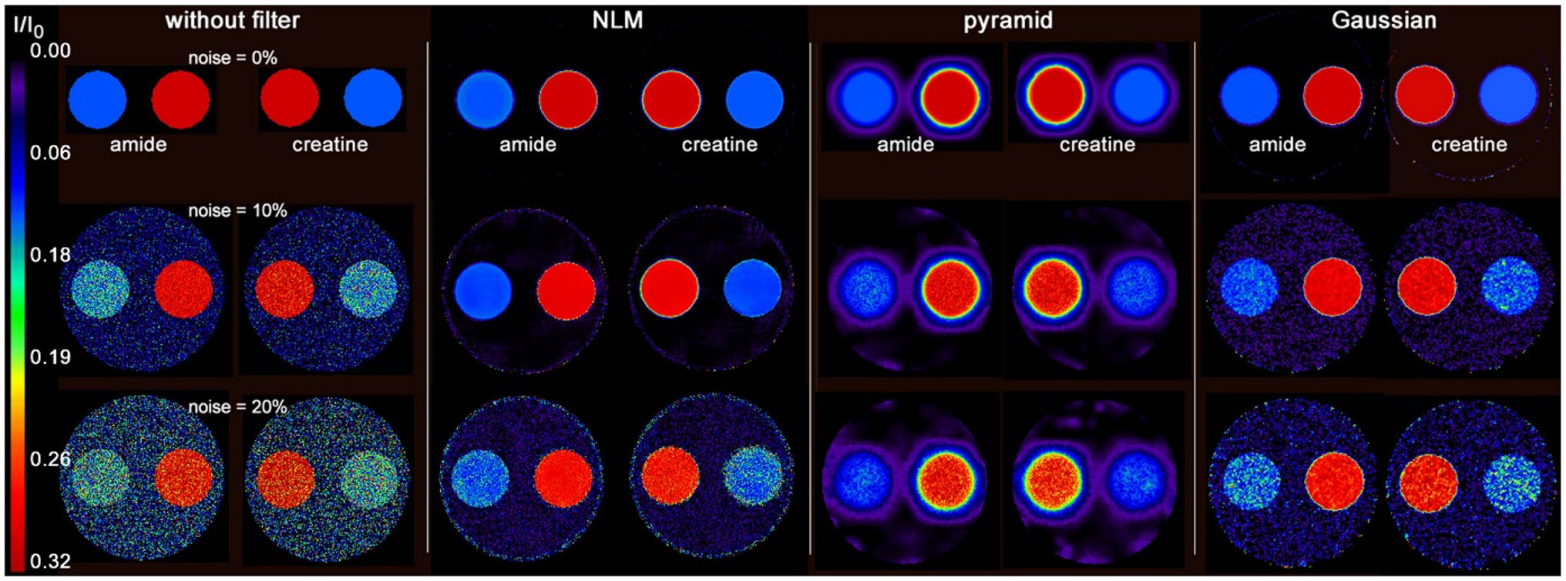
Lorentzian-based amplitude maps of the amide and creatine pools of phantom #1 at three different noise levels resulting from the three approaches: without filtering, with NLM filter, and with pyramidal approach. In addition, the results of a Gaussian filter are displayed

Figure 5 displays the parametric images of amide and creatine at three different noise levels obtained from the three approaches: without filter, with the NLM filter, and with the pyramidal method. As expected, the parameter maps generated without filtering are very noisy at low SNR. NLM and pyramid approach substantially reduce noise in amplitude maps. While NLM preserves the appearance of the phantom, the pyramid method results in a noticeable smoothing of the edges. The results of a 3 × 3 Gaussian filter are shown in Fig. 5 on the right, just to show the difference from the NLM filter. The Gaussian filter produces less noise than no filtering and preserves edges better than the pyramid approach. However, the NLM produces more homogeneous CEST results with less smoothing than the Gaussian filter.

**Fig. 6:**
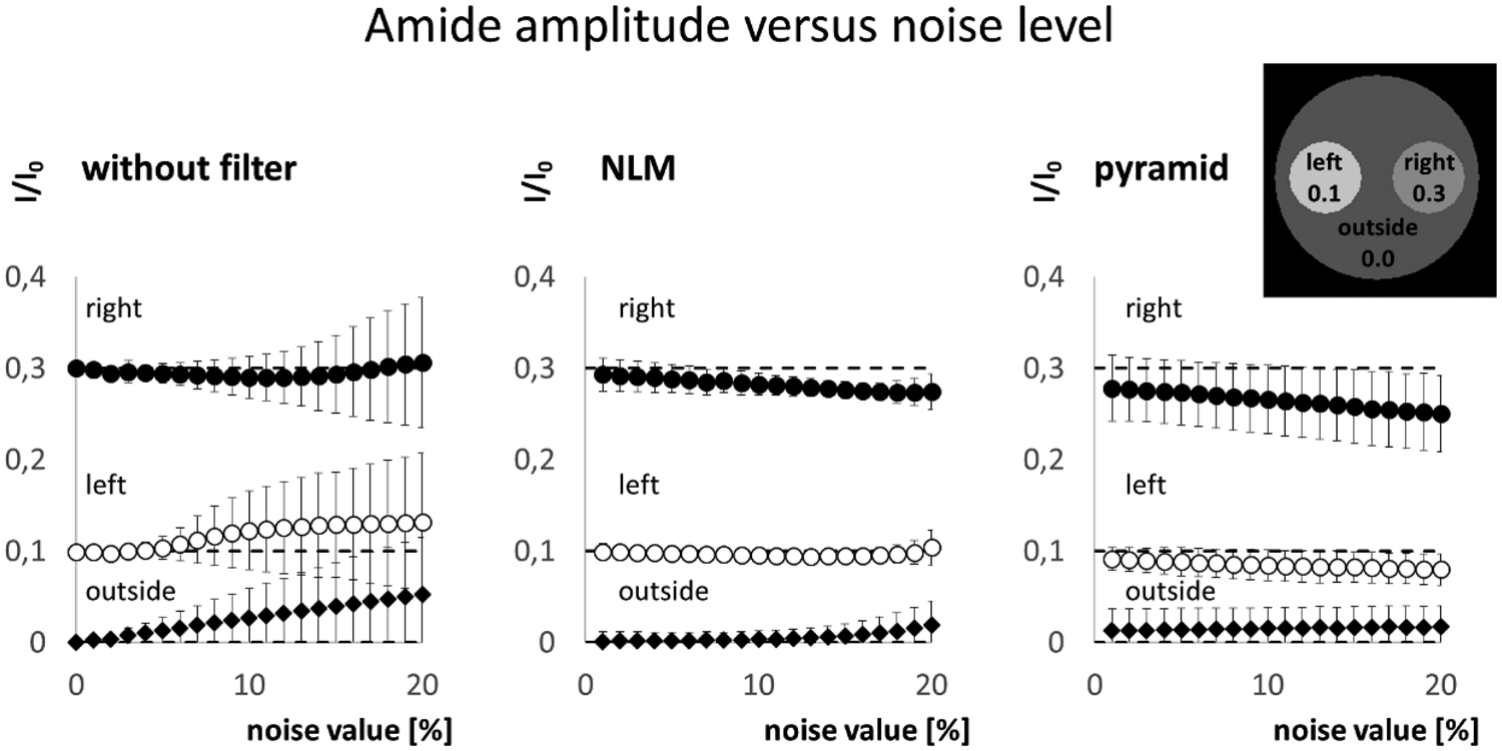
Amide amplitude versus noise level resulting from the three different analysis methods: without filtering, with NLM filter and with pyramidal approach

Figure 6 shows the mean amide amplitude versus noise levels. The dashed lines represent the ground truth amplitude that is 0.3 in the left inner circle, 0.1 in the right inner circle, and 0.0 in the outer region. When no filter was applied, the amplitudes were fitted accurately only up to a noise level of about 5%. At higher noise levels, the fitted amide amplitudes deviate from the ground truth and exhibit increased standard deviations. Interestingly, even in the outer region where no amide pool is present, the fit yielded a distinct amide peak. The erroneous amide peak at low SNR occurs even when the pyramidal approach was used for fitting. Nevertheless, the deviation from the ground truth and the standard deviation within the regions are considerably lower. Lastly, the NLM filtered approach leads to the most stable results with overall lowest standard deviations.

**Fig. 7:**
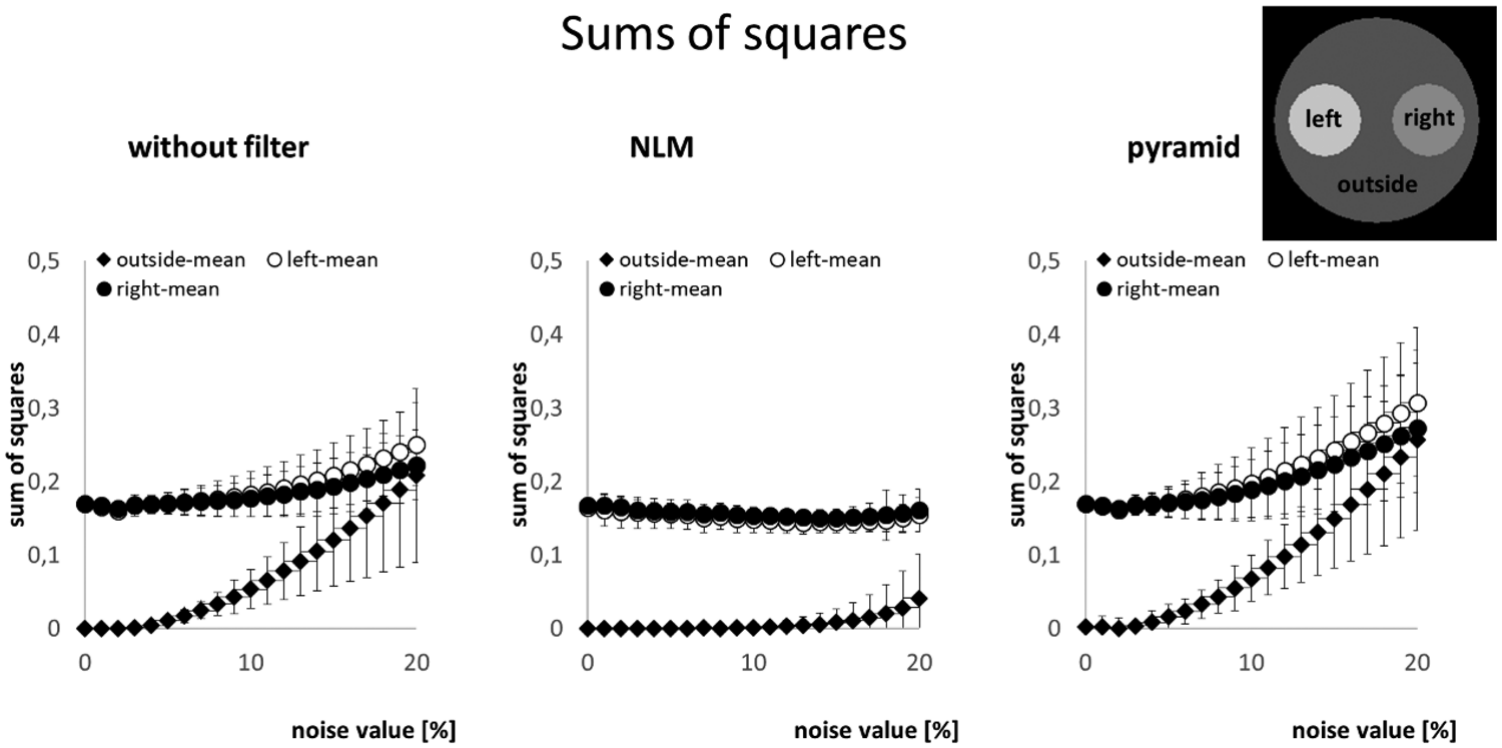
Sum of squares within the three regions in the amide amplitude images versus noise level

Figure 7 shows the mean of the sum of squares of the three regions within the parametric amide images as a quality measure of the fitting process. For the approach without filtering and the pyramidal approach, the quality of the fit is roughly comparable. As the noise level increases, the sum of squares increases. In addition, the standard deviation increases within the three regions, indicating a greater dispersion of results. When using the NLM filter, the fitting process appears to be most stable since only a slight increase in the sum of squares and standard deviations can be observed even at low SNR.

**Fig. 8:**
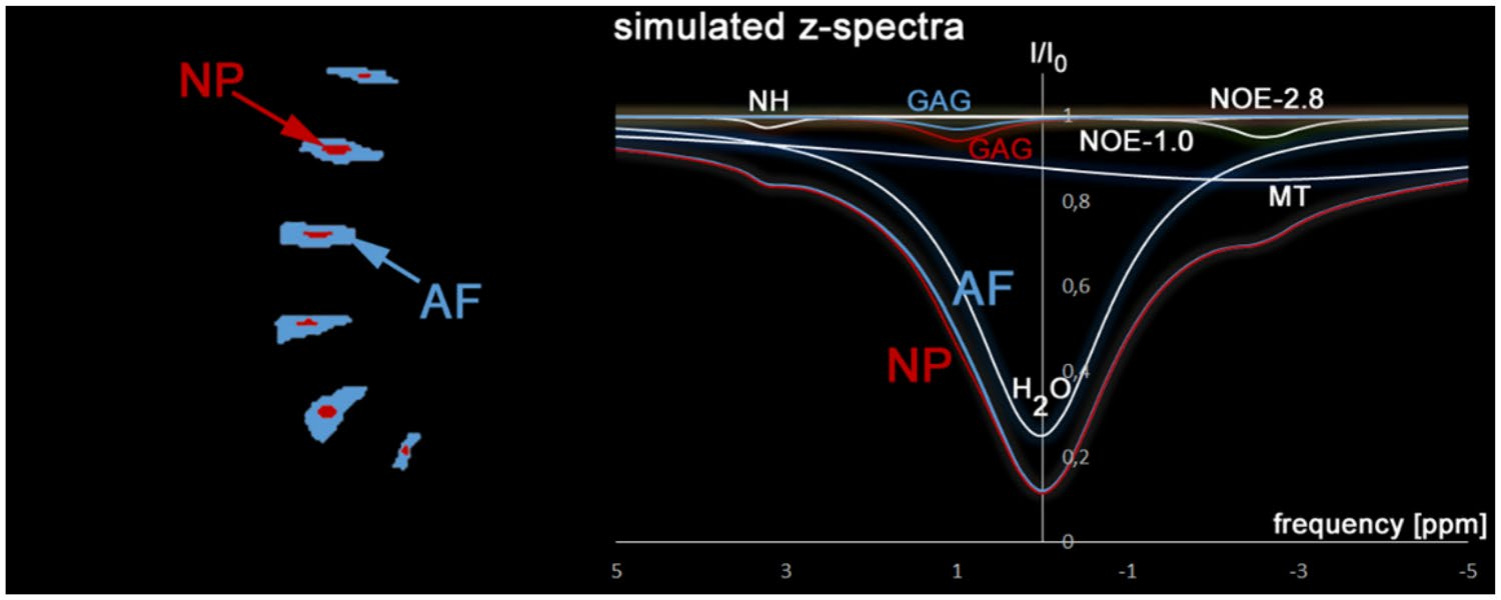
Phantom 2 shows a structure of the intervertebral discs of the lumbar spine. Two areas are defined within each disc: Nucleus pulposus (NP) and Annulus fibrosus (AF). On the right, the simulated spectra of NP (red) and AF (blue) including the individual CEST components (H2O, GAG, MT, NOE-1.0, NOE-2.8, NH) are shown. The individual GAG components of NP (red) and AF (blue) are displayed separately. All other components were held constant in both regions

In phantom 2, which shows the structure of the IVDs of the lumbar spine, a different composition of CEST metabolites was chosen (see Table 2). Figure 8 shows the structure of phantom 2 and exemplary z-spectra for the NP (red) and AF (blue) regions. The right side shows the z-spectra with their individual components. Of note is the small change in GAG (OH) amplitude, resulting in a negligible difference in the overall AF and NP z-spectra (see Fig. 8). Of course, analyzing such small differences requires an effective fitting algorithm especially in the presence of increased image noise.

**Fig. 9:**
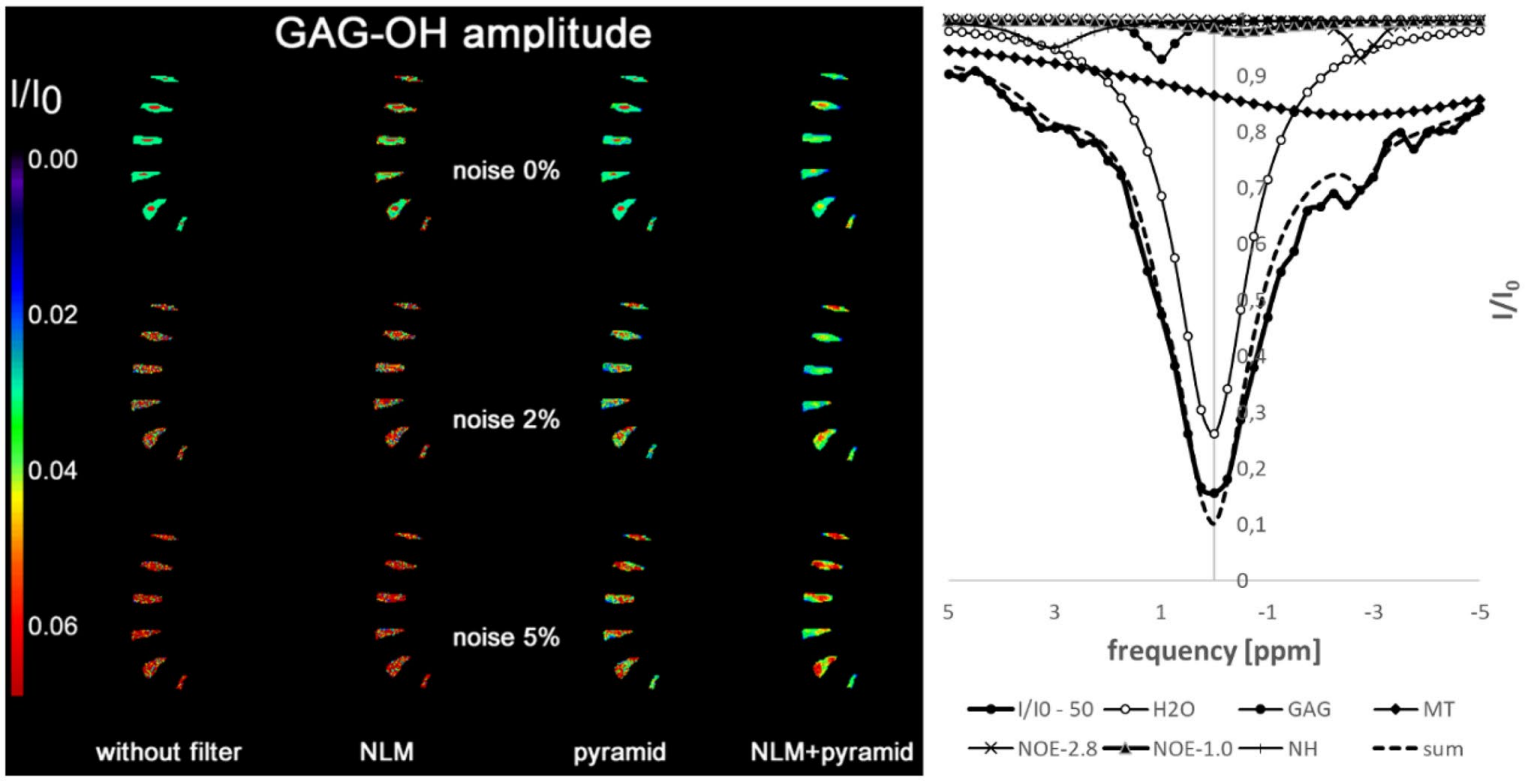
GAG-OH amplitude images of phantom 2 at noise levels 0%, 2% and 5% resulting from the four methods: without filtering, with NLM filter, with pyramidal approach, and with a combined approach of NLM and pyramidal method. Using NLM, pyramidal approach, and combined method, AF and NP are just visible at a noise level of 2%. High noise levels make stable detection of GAG impossible. On the right, an example z-spectrum at a noise level of 5% of the NP including the fitting results is shown. The dashed line represents the fit as sum of all Lorentzian components

Figure 9 shows the GAG-OH amplitude images at three different noise levels obtained from the four methods: without filtering, with NLM filter, with pyramidal approach, and with a combined approach with NLM and pyramidal method. In addition, an example of a z-spectrum with a noise level of 5% of the NP and the results of the fit are shown on the right. Since the GAG amplitude is low, 0.03 for AF and 0.06 for NP (see Table 2), and the regions are very small, the image noise has a strong influence on the GAG results. While at low noise levels of 2% the NP and AF regions are still visible in the GAG amplitude images, the methods fail at higher noise levels. It is worth noting that the combination of NLM and pyramid approach leads to the best results for the GAG amplitude images at a noise level of 2% and to overall lower standard deviations within the AF and NP regions. In general, the results obtained in phantom 2 show that stable detection of CEST components with very low amplitudes and amplitude differences associated with small spatial regions requires CEST data with high signal-to-noise ratio.

**Fig. 10:**
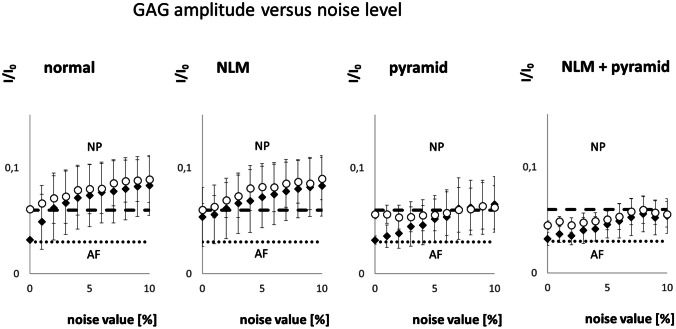
GAG amplitude versus noise level resulting from the three different analysis methods: without filtering, with NLM filter and with pyramidal approach. The dotted line represents the ground truth in AF, the dashed line in NP. Due to the small difference in amplitudes and the small regions of AF and NP, discrimination is only possible with low noise. The combination of NLM and pyramidal approach leads to smaller standard deviations within the AF and NP regions

The visual results of the GAG amplitude images are reflected in the analysis of the mean amplitudes of AF and NP in Fig. 10, where the regional mean and standard deviations are shown. Only at low noise levels, the amplitudes of GAG are close to the ground truth marked by the dotted and dashed lines. As noise increases, the AF and NP results strongly deviate from the ground truth and are, therefore, indistinguishable from each other.

### Phantom measurement

**Fig. 11:**
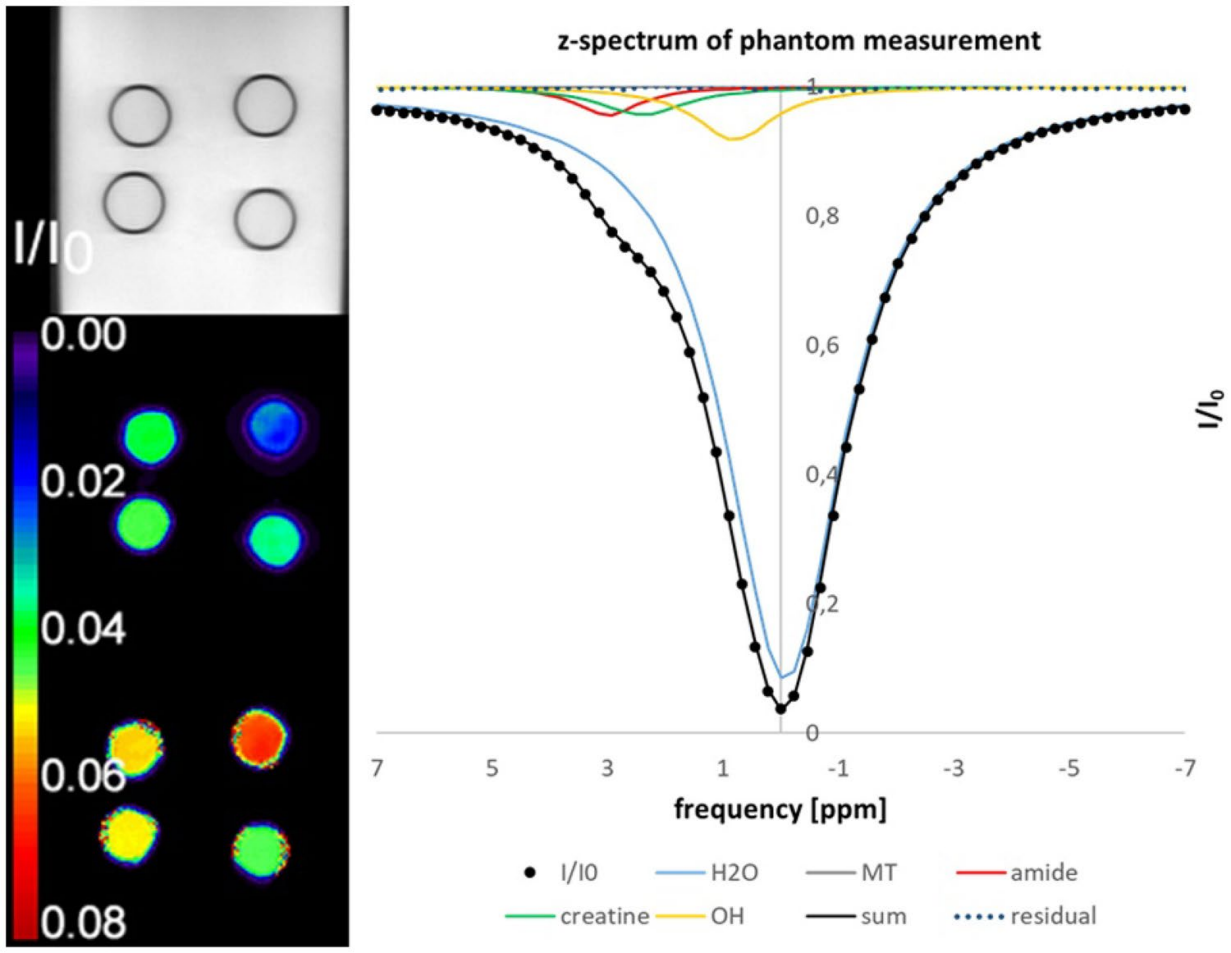
CEST measurement of the phantom containing NAD and creatine. The left side shows, from top to bottom, the I_0_ image, the NAD map and the creatine map. On the right, the z-spectrum of the upper right tube is shown with the fitting results for the sum and the individual components as well as the residual. Data were filtered with NLM before Lorentzian fitting

As the next step in the software evaluation process, the results of the real phantom measurement are presented. Figure 11 shows the results of the in-vitro CEST experiments. The Lorentzian model function fits the measured z-spectrum very well as proven by the residual. The different concentrations are reflected in the parametric images of NAD and Cr. The variation seen in tubes with the same concentrations may be due to B1 inhomogeneity effects. In addition, the mixing of the phantom with not fully dissolved substances was imperfect because the concentrations were close to the maximum dissolvable. The variations for the same concentrations were 17% for NAD and 8% for creatine.

### In-vivo study

**Fig. 12:**
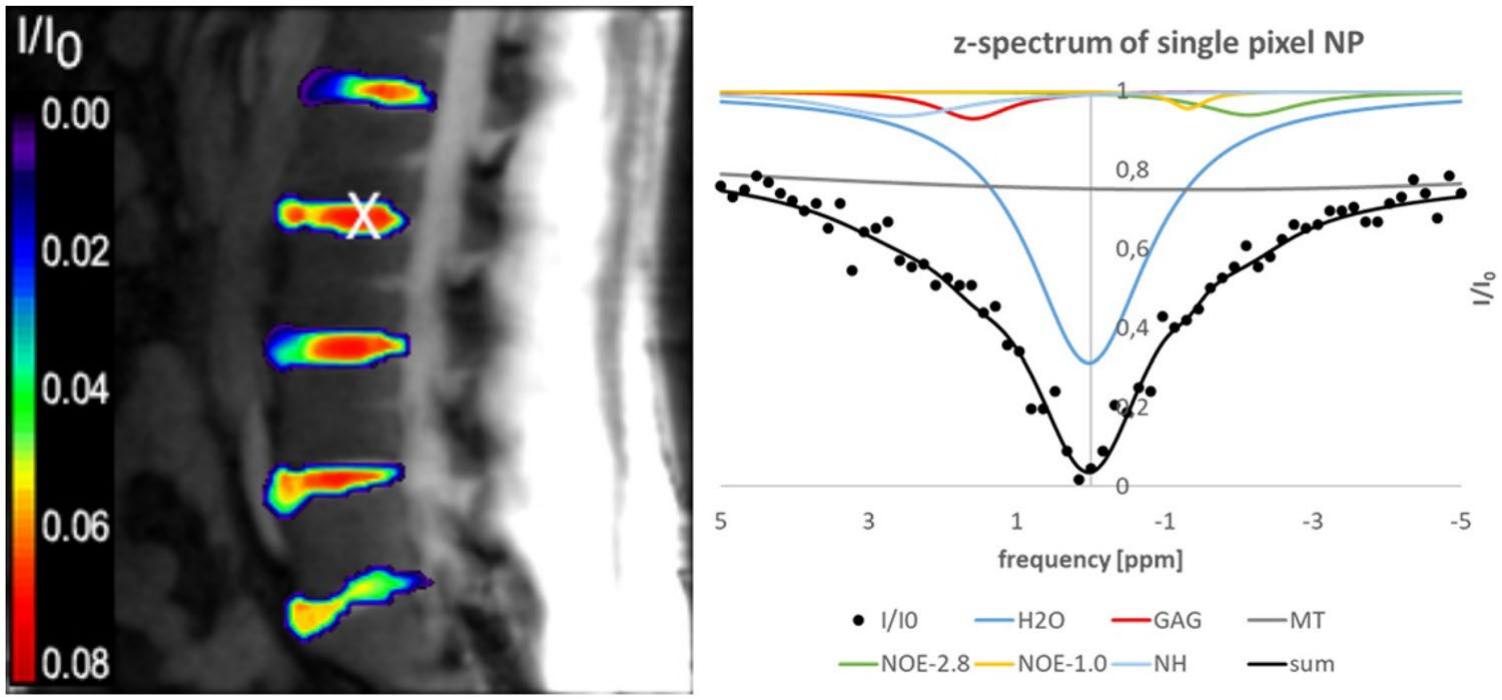
In vivo GAG amplitude image superimposed on an anatomical image. On the right, the z-spectrum of a single pixel is shown at the pixel position marked by the white cross. The individual Lorentzian lines and the sum of all components (black line) are displayed

The last step of the software evaluation was an in vivo study. Figure 12 shows results of the in-vivo CEST examination of the lumbar spine of a healthy subject. On the left, the GAG amplitude image is superimposed on the unsaturated I_0_ image. The z-spectrum and fitted Lorentzian peaks generated for a single pixel are shown on the right. The position of the pixel is displayed as an X-mark on the anatomical image. The black line indicates the fitted curve of the measured data. Based on the results of the digital phantom 2, the analysis was performed by the combined method with NLM filter and pyramid approach. As expected, the central NP regions of the IVDs show higher GAG amplitudes than the outer AF regions.

## Discussion

In this study, we introduce the new CEST analysis software "calf", which evaluates z-spectra by fitting Lorentzian lines and features various denoising algorithms. The software has a convenient user interface and is easy to use for the analysis of CEST data. For this purpose, we have defined a general functional consisting of the sum of the squared differences between measured data and model function. A special nonlinear least squares algorithm is used to minimize the functional, allowing robust determination of the Lorentzian line shape parameter estimates even for sparsely sampled data with low SNR. The advantage of this novel approach is that the analysis can be performed on the original low-sampling CEST data without the need for potentially defective upsampling. From the signal theory, it is known that interpolation of data can lead to deflections or oscillations [33]. These may negatively impact the CEST analysis by introducing errors in the parameter estimates. This problem can be circumvented by processing the unmodified original measurement data directly, as it is the case with our proposed algorithm. In addition, automatic frequency shift correction by pre-fitting eliminates the need for B_0_ map acquisition to correct for B_0_ field inhomogeneity. Further, pyramidal approach similar to the IDEAL method described in [12] and a non local means noise filter were implemented and tested for CEST processing. Both approaches serve to improve data quality through noise reduction, which is a relevant factor in the analysis of z-spectra. Taken together, the proposed analysis methods address the typical challenges of CEST imaging: a low sampling rate in the frequency direction, limited SNR, overlapping peaks from different CEST pools that require integration of prior knowledge into fitting process, and frequency shifts caused by B_0_ field inhomogeneity.

Validation of the proposed processing pipeline was performed in-silico, in-vitro and in-vivo. First, to test the accuracy of the developed methods, various digital phantoms were created. Different amounts of noise were added to the images in order to better mimic a series of in-vivo conditions. Subsequently the proposed analysis methods were tested using a phantom consisting of nicotinamide and creatine solutions. Furthermore, an in-vivo study was performed on the lumbar spine of a healthy subject to demonstrate the performance of the developed methods for analysis of in-vivo data.

The digital phantom 1 consisted of large compartments containing large amounts of amide and creatine. Although this configuration promises a simple CEST analysis, when the signal noise is high, the fitting appears complicated due to the occurrence of large pseudo noise peaks. The application of NLM noise filter or the pyramidal approach delivers stable results anyway, even the pyramidal approach leads to a relevant smoothing of the spatial structures.

Phantom 2 was modeled on the structure of a lumbar spine, with small areas defined by the annulus fibrosus and nucleus pulposus. As in in-vivo conditions, the amount of GAG was only slightly different, resulting in very similar z-spectra. Therefore, the analysis process in the presence of image noise is a challenge that requires a high signal-to-noise ratio to obtain stable results. A combination of NLM filter and pyramidal approach showed the best results in this regime.

As a next step to validate the developed algorithms, data from a phantom with different amounts of creatine and nicotinamide were examined and analyzed. The CEST data of the phantom could be reliably analyzed with respect to the pools of amide and creatine. The fit of the z-spectra was good with very small distance squares.

To validate our multi-pool fitting algorithm with real in vivo CEST data, the examination of the lumbar spine of a healthy subject was recorded and analyzed. Although ground truth is understandably not available, the quality of the analysis with our algorithms can be described as good. The geometric shape with higher GAG amplitudes within NP areas is realistic and comparable to previous studies [34]. It is noteworthy here that the proposed analysis does not require external corrections of B0-induced frequency shifts and time-consuming measurement methods such as WASSR. Upsampling of the z-spectra was also not necessary.

Although quantitative CEST may be used in the future to determine labile proton concentration and the corresponding exchange rate, it is not currently used for in-vivo measurements because of high complexity of biological tissues, which contain a large variety of metabolites, proteins and macromolecules. The contributions from all these potential CEST pools cannot be separated by the conventional MTR_asym_ analysis. Moreover, the in-vivo measured MTR_asym_ values are often significantly affected by the semisolid MT asymmetry, NOE effects and B_0_ shifts. As demonstrated in this and previous studies, the application of Lorentzian fitting improves the interpretation of the individual CEST effects. This might be particularly advantageous when analyzing CEST data acquired at high pre-saturation power levels that create a stronger overlap between different CEST peaks and enhance the confounding direct spillover effect.

The pyramidal method, which is similar to the IDEAL approach [12], can lead to regionally varying inaccuracies, especially in highly heterogenous tissues that are present within the same image. Moreover, the downsampling and upsampling process may introduce further image artifacts such as aliasing and staircase artifacts. Therefore, we implemented the edge-preserving spatial filtering method NLM to reduce the image noise. As shown in our study, a combination of the NLM and the pyramidal approach can further improve CEST analysis.

There are already other software tools for processing CEST data. Most of them are suitable for advanced users. QuantiCEST is part of the large Quantiphyse toolbox and uses sophisticated Bayesian analysis methods for quantitative CEST analysis [35, 36]. QuantiCEST has been developed in Python and therefore requires an initial installation of the Python environment. Although QuantiCEST is easy to use, its detailed and sophisticated features make it more suitable for advanced CEST experts. Cest_eval from the cest-sources project is a very sophisticated CEST tool that uses Lorentzian fitting, but also implements quantitative methods such as MTRRex or AREX [37, 38]. As it is developed in Matlab, it is aimed at CEST professionals with Matlab skills. BayCEST uses the Bayesian approach and has been developed as part of the extended FSL suite for advanced analysis of functional brain imaging data [34, 39]. FSL runs under the Linux operating system and is a very comprehensive brain suite tool. Therefore, BayCEST is intended for specialists only. MITK is a large and sophisticated medical imaging platform and medical imaging toolkit for the development of interactive medical imaging software. MITK CEST is a module implemented on top of MITK [40]. It enables MTR_asym_, Lorentzian fitting, and WASABI [41]. Due to the complex nature of MITK, it is also intended for advanced users. There are other tools available for the analysis of CEST data, but most of them are written in Matlab or Python and are intended for use only by medical imaging scientists.

In contrast, calf has been developed with the intention of being easy to use, not only for experts in CEST MRI. Much effort has been put into usability and the graphical user interface (GUI). The GUI is that of a standard Microsoft Windows application, where even less experienced users can get along. Additional calf can be started by simply double-clicking on the application, even without software installation.

Recent studies by Cohen et al. and Perlman et al. have demonstrated the potential of deep learning for CEST imaging, but may not be accessible or transparent to a broader, non-technical audience [42, 43]. However, we see the potential to incorporate AI-based methods into our system in the future to improve the performance and efficiency of the analysis.

The main purpose of our study was to introduce a free software for an easy and reliable analysis of CEST data. In the future, further studies need to be conducted comparing the implemented algorithms with other existing methods, e.g., in terms of noise reduction of z-spectra and other techniques to improve the stability of the CEST results such as image registration or B_0_ and B_1_ corrections [44–46].

In summary, we have developed a novel tool for Lorentzian analysis of CEST data, which uses the edge-preserving non local mean noise filter and pyramidal approach to provide robust quantification of the CEST MRI effects in a multipool system. The proposed methods were validated using in-silico, in-vitro and in-vivo CEST data. The algorithms were implemented in easy-to-use software that runs on the Microsoft Windows operating system and will be made available to the CEST research community.

## Data Availability

The data that support the findings of this study are available on request from the corresponding author.

## References

[1] Sherry AD, Woods M (2008). Chemical exchange saturation transfer contrast agents for magnetic resonance imaging. Annual review of biomedical engineering.

[2] van Zijl PC, Yadav NN (2011). Chemical exchange saturation transfer (CEST): what is in a name and what isn’t?. Magnetic resonance in medicine.

[3] Ward KM, Aletras AH, Balaban RS (2000). A new class of contrast agents for MRI based on proton chemical exchange dependent saturation transfer (CEST). Journal of magnetic resonance.

[4] Jones KM, Pollard AC, Pagel MD (2018). Clinical applications of chemical exchange saturation transfer (CEST) MRI. J Magn Reson Imaging.

[5] Cai K (2015). CEST signal at 2 ppm (CEST@2 ppm) from Z-spectral fitting correlates with creatine distribution in brain tumor. NMR in biomedicine.

[6] Walker-Samuel S (2013). In vivo imaging of glucose uptake and metabolism in tumors. Nature medicine.

[7] Sun PZ, Zhou J, Sun W, Huang J, van Zijl PC (2007). Detection of the ischemic penumbra using pH-weighted MRI. Journal of cerebral blood flow and metabolism: official journal of the International Society of Cerebral Blood Flow and Metabolism.

[8] Jia G (2011). Amide proton transfer MR imaging of prostate cancer: A preliminary study. J Magn Reson Imaging.

[9] Chen LQ (2014). Evaluations of extracellular pH within in vivo tumors using acidoCEST MRI. Magnetic resonance in medicine.

[10] Sagiyama K (2014). In vivo chemical exchange saturation transfer imaging allows early detection of a therapeutic response in glioblastoma. Proc Natl Acad Sci USA.

[11] Zaiss M, Schmitt B, Bachert P (2011). Quantitative separation of CEST effect from magnetization transfer and spillover effects by Lorentzian-line-fit analysis of z-spectra. Journal of magnetic resonance.

[12] Zhou IY, Wang E, Cheung JS, Zhang X, Fulci G, Sun PZ (2017). Quantitative chemical exchange saturation transfer (CEST) MRI of glioma using Image Downsampling Expedited Adaptive Least-squares (IDEAL) fitting. Sci Rep..

[13] Breitling J, Deshmane A, Goerke S, Korzowski A, Herz K, Ladd ME, Scheffler K, Bachert P, Zaiss M (2019). Adaptive denoising for chemical exchange saturation transfer MR imaging. NMR Biomed..

[14] Kim M, Gillen J, Landman BA, Zhou J, van Zijl PC (2009). Water saturation shift referencing (WASSR) for chemical exchange saturation transfer (CEST) experiments. Magn Reson Med..

[15] Müller-Lutz A, Ljimani A, Stabinska J, Zaiss M, Boos J, Wittsack HJ, Schleich C (2018). Comparison of B_0_ versus B_0_ and B_1_ field inhomogeneity correction for glycosaminoglycan chemical exchange saturation transfer imaging. MAGMA.

[16] Wei W, Jia G, Flanigan D, Zhou J, Knopp MV (2014). Chemical exchange saturation transfer MR imaging of articular cartilage glycosaminoglycans at 3 T: Accuracy of B0 Field Inhomogeneity corrections with gradient echo method. Magn Reson Imaging.

[17] Schreiner MM, Zbýň Š, Schmitt B, Weber M, Domayer S, Windhager R, Trattnig S, Mlynárik V (2016). Reproducibility and regional variations of an improved gagCEST protocol for the in vivo evaluation of knee cartilage at 7 T. MAGMA.

[18] Heo HY, Zhang Y, Lee DH, Hong X, Zhou J. (2016) Quantitative assessment of amide proton transfer (APT) and nuclear overhauser enhancement (NOE) imaging with extrapolated semi-solid magnetization transfer reference (EMR) signals: Application to a rat glioma model at 4.7 Tesla. Magn Reson Med 75(1):137–49 10.1002/mrm.25581 PMC4561043 25753614

[19] Geades N, Hunt BAE, Shah SM, Peters A, Mougin OE, Gowland PA (2017). Quantitative analysis of the z-spectrum using a numerically simulated look-up table: Application to the healthy human brain at 7T. Magn Reson Med.

[20] Zhang XY, Wang F, Afzal A, Xu J, Gore JC, Gochberg DF, Zu Z. (2016) A new NOE-mediated MT signal at around -1.6ppm for detecting ischemic stroke in rat brain. Magn Reson Imaging 34(8):1100–1106 10.1016/j.mri.2016.05.002 PMC4993652 27211260

[21] Zhang J, Zhu W, Tain R, Zhou XJ, Cai K (2018). Improved Differentiation of Low-Grade and High-Grade Gliomas and Detection of Tumor Proliferation Using APT Contrast Fitted from Z-Spectrum. Mol Imaging Biol.

[22] Krikken E, Khlebnikov V, Zaiss M, Jibodh RA, van Diest PJ, Luijten PR, Klomp DWJ, van Laarhoven HWM, Wijnen JP. (2018) Amide chemical exchange saturation transfer at 7 T: a possible biomarker for detecting early response to neoadjuvant chemotherapy in breast cancer patients. Breast Cancer Res. Jun 14;20(1):51 10.1186/s13058-018-0982-2 PMC6001024 29898745

[23] van der Veen JW, de Beer R, Luyten PR, van Ormondt D. (1988) Accurate quantification of in vivo 31P NMR signals using the variable projection method and prior knowledge. Magn Reson Med Jan;6(1):92–98 10.1002/mrm.1910060111 3352510

[24] Vanhamme L, van den Boogaart A, Van Huffel S. (1997) Improved method for accurate and efficient quantification of MRS data with use of prior knowledge. J Magn Reson Nov;129(1):35–43 10.1006/jmre.1997.1244 9405214

[25] Dennis John, Gay David, Welsch Roy (1981). Algorithm 573: An Adaptive Nonlinear Least-Squares Algorithm. ACM Transactions on Mathematical Software.

[26] Dennis JE, Schnabel RB (1983). Numerical Methods for Unconstrained Optimisation and Nonlinear Equations.

[27] Butcher JC, Jackiewicz Z, Mittelmann HD (1997). A nonlinear optimization approach to the construction of general linear methods of high order. Journal of Computional and Applied Mathematics.

[28] https://www.netlib.org/ (01/26/2023)

[29] Gill PE, Murray W, Wright MH (1988). Practical Optimization.

[30] Buades A (2005). A non-local algorithm for image denoising. Computer Vision and Pattern Recognition.

[31] https://opencv.org/ (01/26/2023)

[32] Kim M, Gillen J, Landman BA, Zhou J, van Zijl PC (2009). Water saturation shift referencing (WASSR) for chemical exchange saturation transfer (CEST) experiments. Magn Reson Med.

[33] Meijering EA (2002). Chronology of Interpolation: From Ancient Astronomy to Modern Signal and Image Processing. Proc. IEEE.

[34] Müller-Lutz A, Schleich C, Schmitt B, Antoch G, Matuschke F, Quentin M, Wittsack HJ, Miese F (2016). Gender, BMI and T2 dependencies of glycosaminoglycan chemical exchange saturation transfer in intervertebral discs. Magn Reson Imaging.

[35] Chappell MA, Donahue MJ, Tee YK, Khrapitchev AA, Sibson NR, Jezzard P, Payne SJ (2013). Quantitative Bayesian model-based analysis of amide proton transfer MRI. Magn Reson Med..

[36] Croal PL, Msayib Y, Ray KJ, Craig M, Chappell M. QuantiCEST: Bayesian Model-based Analysis of CEST MRI. June 2018 Conference: ESMRMB-ISMRM Joint Annual Meeting 2018, Paris, France

[37] Zaiss M, Xu J, Goerke S, Khan IS, Singer RJ, Gore JC, Gochberg DF, Bachert P (2014). Inverse Z-spectrum analysis for spillover-, MT-, and T1 -corrected steady-state pulsed CEST-MRI–application to pH-weighted MRI of acute stroke. NMR Biomed..

[38] Windschuh J, Zaiss M, Meissner JE, Paech D, Radbruch A, Ladd ME, Bachert P (2015). Correction of B1-inhomogeneities for relaxation-compensated CEST imaging at 7 T. NMR Biomed..

[39] Chappell M, Groves A, Whitcher B, Woolrich M (2009). Variational Bayesian Inference for a Nonlinear Forward Model. IEEE Transactions on Signal Processing.

[40] Debus C, Floca R, Ingrisch M, Kompan I, Maier-Hein K, Abdollahi A, Nolden M (2019). MITK-ModelFit: A generic open-source framework for model fits and their exploration in medical imaging - design, implementation and application on the example of DCE-MRI. BMC Bioinformatics..

[41] Schuenke P, Windschuh J, Roeloffs V, Ladd ME, Bachert P, Zaiss M (2017). Simultaneous mapping of water shift and B1 (WASABI)-Application to field-Inhomogeneity correction of CEST MRI data. Magn Reson Med..

[42] Cohen O, Yu VY, Tringale KR, Young RJ, Perlman O, Farrar CT, Otazo R (2023). CEST MR fingerprinting (CEST-MRF) for brain tumor quantification using EPI readout and deep learning reconstruction. Magn Reson Med..

[43] Perlman O, Zhu B, Zaiss M, Rosen MS, Farrar CT (2022). An end-to-end AI-based framework for automated discovery of rapid CEST/MT MRI acquisition protocols and molecular parameter quantification (AutoCEST). Magn Reson Med..

[44] Breitling J, Deshmane A, Goerke S, Korzowski A, Herz K, Ladd ME, Scheffler K, Bachert P, Zaiss M (2019). Adaptive denoising for chemical exchange saturation transfer MR imaging. NMR Biomed.

[45] Müller-Lutz A, Schleich C, Pentang G, Schmitt B, Lanzman RS, Matuschke F, Wittsack HJ, Miese F (2015). Age-dependency of glycosaminoglycan content in lumbar discs: A 3t gagcEST study. J Magn Reson Imaging.

[46] Glang F, Deshmane A, Prokudin S, Martin F, Herz K, Lindig T, Bender B, Scheffler K, Zaiss M (2020). DeepCEST 3T: Robust MRI parameter determination and uncertainty quantification with neural networks-application to CEST imaging of the human brain at 3T. Magn Reson Med.

